# *Burkholderia pseudomallei* Laboratory Exposure, Arizona, USA

**DOI:** 10.3201/eid2905.221865

**Published:** 2023-05

**Authors:** Lisa J. Speiser, Erin H. Graf, Maria Teresa Seville, Kai Singbartl, Mary L. Dalton, Denise Harrington, Melissa Kretschmer, Marina Kuljanin, Karen Zabel, Rebecca Sunenshine, Irene Ruberto, Heather Venkat, Thomas E. Grys

**Affiliations:** Mayo Clinic Hospital, Phoenix, Arizona, USA (L.J. Speiser, E.H. Graf, M.T. Seville, K. Singbartl, M.L. Dalton, D. Harrington, T.E. Grys);; Maricopa County Department of Public Health, Phoenix (M. Kretschmer, M. Kuljanin, K. Zabel, R. Sunenshine);; Arizona Department of Health Services, Phoenix (I. Ruberto, H. Venkat);; Centers for Disease Control and Prevention, Atlanta, Georgia, USA (H. Venkat)

**Keywords:** *Burkholderia pseudomallei*, bacteria, melioidosis, laboratory exposure, Arizona, United States

## Abstract

We describe an incidental *Burkholderia pseudomallei* laboratory exposure in Arizona, USA. Because melioidosis cases are increasing in the United States and *B. pseudomallei* reservoirs have been discovered in the Gulf Coast Region, US laboratory staff could be at increased risk for *B. pseudomallei* exposure.

*Burkholderia pseudomallei* bacterium, the causative agent of melioidosis, is endemic to Australia and Thailand. However, the US Centers for Disease Control and Prevention (CDC) recently discovered positive environmental samples in the Gulf Coast Region of Mississippi, USA, when investigating 2 melioidosis cases (*1*). In 2021, 4 melioidosis cases in the United States were found to be caused by imported aromatherapy spray contaminated with *B. pseudomallei* (*2*). Because melioidosis cases are increasing in the United States, laboratory staff potentially are at risk for *B. pseudomallei* exposure. In nonendemic areas, laboratory staff are unfamiliar with *B. pseudomallei*, and the bacterium commonly is misidentified. As occurred with the 2 melioidosis cases related to aromatherapy products (*2*), matrix-assisted laser desorption/ionization time-of-flight (MALDI-TOF) libraries often misidentify *B. pseudomallei* as *B. thailandensis*. We describe an incidental *B. pseudomallei* laboratory exposure in Arizona, USA.

In mid-January 2021, the microbiology laboratory at Mayo Clinic Arizona (Phoenix, AZ, USA) identified *Burkholderia* species growing from an intraoperative periaortic swab sample obtained from a 58-year-old man with a mycotic aneurysm (*3*). Results of routine Gram stains of all specimens were negative. Aerobic cultures revealed pinpoint growth on sheep blood and chocolate agars, but not on MacConkey agar, after 18 hours. Staff performed Gram stain of the colonies, which revealed gram-negative rods. The technologist suspected an atypical *Pseudomonas* species and, on an open benchtop, performed oxidase testing, with positive results, and spot indole testing, with negative results. MALDI-TOF mass spectrometry provided an unvalidated *B. thailandensis* identification. Because of concerns that the unvalidated result could suggested *B. pseudomallei*, staff performed slide catalase testing on a fresh subculture per the Laboratory Response Network Sentinel Level Clinical Laboratory Protocol (*4*). The catalase reaction was negative, which was inconsistent with *Burkholderia* species. The laboratory then sent the isolate to the Mayo Clinic reference laboratory (Rochester, MN, USA) for definitive identification. By using a laboratory-developed MALDI-TOF database that was considered unvalidated, the reference laboratory presumptively identified the isolate as *B. pseudomallei.* The Minnesota Public Health Laboratory confirmed *B. pseudomallei* through molecular and biochemical methods. Repeat catalase testing found the isolate to be slide catalase–negative but weakly tube catalase–positive. The isolate was transferred to CDC for antimicrobial-susceptibility testing, which demonstrated a typical susceptibility profile to trimethoprim/sulfamethoxazole, doxycycline, amoxicillin/clavulanic acid, and ceftazidime. *B. pseudomallei* growth was eventually observed on both MacConkey and colistin nalidixic acid agars and on all anaerobic, mycobacterial, and fungal culture media. *B. pseudomallei* is a Select Agent, thus, the Federal Select Agent Program was notified, and all cultures were destroyed within 7 days of definitive identification. 

Because of initial lack of clinical suspicion for *B. pseudomallei*, we evaluated clinical staff for exposure. We identified 30 employees who had possible exposure. We assessed each employee for exposure risk, as previously described (*5*), and identified 3 employees who were exposed in the microbiology laboratory: 1 high-risk and 2 low-risk exposures. The employee with high-risk exposure had a predisposing condition and performed an aerosolizing procedure outside of the biologic safety cabinet by subjecting the specimen to MALDI-TOF mass spectrometry without first inactivating it. The 2 employees with low-risk exposures participated in close inspection of the open plate growing *B. pseudomallei* outside of the biologic safety cabinet.

Laboratory-acquired melioidosis is extremely rare. Reports of 2 prior laboratory-acquired melioidosis cases in the United States have been published (*6*,*7*), but none have been reported since 1981. As for the high-risk exposure we describe, both published cases were attributed to aerosol exposure (*6*,*7*). *B. mallei* is considered to have greater potential for laboratory infection than *B. pseudomallei* (*8*).

In animal models, postexposure prophylaxis (PEP) has been shown to effectively prevent acute melioidosis if administered within 24 hours of exposure (*9*). However, PEP fails to prevent latent or persistent infection (*10*); nonetheless, consensus recommendations are to offer PEP to all employees with high- and low-risk incidents, regardless of their predisposing risk for melioidosis (*5*). After explaining risks versus benefits, we offered the employee with high-risk exposure a 3-week duration of trimethoprim/sulfamethoxazole PEP (*5*,*9*). However, the employee stopped PEP after 1 week because of insomnia; no subsequent PEP was prescribed because the employee stopped PEP without consulting a medical provider. On the basis of guidance from the Maricopa County Department of Public Health, we offered PEP to the employees with low-risk exposures; 1 elected to take doxycycline, and the other declined PEP.

We instructed exposed employees to monitor their temperatures 2 times a day for 21 days and notify the hospital’s occupational health department if symptoms occurred. None of the employees reported symptoms during the monitoring period.

Because *B. pseudomallei* can persist intracellularly for extended periods before causing clinical disease, we requested assistance from the Arizona Department of Health Services, Maricopa County Department of Public Health, and CDC to offer serologic monitoring to the exposed employees; 2 elected to undergo serologic monitoring. After 6 weeks, neither employee seroconverted.

In conclusion, lack of clinical and laboratory suspicion for *B. pseudomallei* resulted in incidental laboratory exposure of 3 employees. US laboratories should remain vigilant for and aware of the growth characteristics associated with *B. pseudomallei* to help avoid occupational exposure.
